# Sociodemographic and Lifestyle Determinants of Adherence to Current Dietary Recommendations and Diet Quality in Middle-Aged Spanish Premenopausal Women

**DOI:** 10.3389/fnut.2022.904330

**Published:** 2022-06-14

**Authors:** Marina Alonso-Cabezas, Marina Pollán, Isabel Alonso-Ledesma, Nerea Fernández de Larrea-Baz, Pilar Lucas, Ángeles Sierra, Adela Castelló, Marina Nieves Pino, Beatriz Pérez-Gómez, Mercedes Martínez-Cortés, Virginia Lope, Emma Ruiz-Moreno

**Affiliations:** ^1^Department of Nutrition and Food Sciences, Faculty of Farmacy, Complutense University of Madrid, Madrid, Spain; ^2^Department of Epidemiology of Chronic Diseases, National Center for Epidemiology, Instituto de Salud Carlos III, Madrid, Spain; ^3^Consortium for Biomedical Research in Epidemiology & Public Health (CIBERESP), Instituto de Salud Carlos III, Madrid, Spain; ^4^Department of Surgery, Medical and Social Sciences, Faculty of Medicine, University of Alcalá, Alcalá de Henares, Madrid, Spain; ^5^Servicio de Prevención y Promoción de la Salud, Madrid Salud, Ayuntamiento de Madrid, Madrid, Spain

**Keywords:** SENC recommendations, premenopause, dietary reference values, healthy diet, dietary guidelines, dietary habits, DDM-Madrid, cross-sectional study

## Abstract

**Background:**

A healthy diet when approaching menopause could prevent some of the symptoms associated with the climacteric. Few studies examine adherence to current healthy dietary recommendations in middle-aged premenopausal women. Our objective was to analyze the diet quality and the adherence to the Spanish Society of Community Nutrition (SENC) dietary recommendations in middle-aged Spanish premenopausal women, and to identify the associated sociodemographic and lifestyle factors.

**Methods:**

This is a cross-sectional study based on 1251 premenopausal women, aged 39–50, who attended to Madrid City Council Medical Diagnostic Center. Women completed an epidemiological and a food frequency questionnaire. Degree of adherence to the SENC recommendations was estimated with a score that evaluated null (0 points) and full (1 point) adherence of each specific recommendation. Associations were explored using an ordinal logistic multivariable regression model.

**Results:**

Regarding food groups, the worst adherence was found for sweets, red/processed meat, olive oil and eggs. Most of the participants exceeded the recommended caloric intake from proteins and fats, and practically all of them showed vitamin D intake deficiency. The overall score ranged from 2 to 12 (out of 15), with a median of 6.0 (interquartile range: 5.0–7.0). Former smokers (OR: 1.38; 95%CI: 1.08–1.78), as well as those with higher educational level (OR_SSecondary_:1.68; 95%CI: 0.97–2.93, OR_University_:1.82; 95%IC: 1.05–3.14), with two or more children (OR: 1.31; 95%IC: 1.00-1.72), with higher caloric intake (OR_>2188.2kcal/day_: 8.22; 95%CI: 6.19–10.92) and with greater physical activity (OR_≥21METS−h/week_: 1.29; 95%CI: 0.95-1.76) showed greater adherence.

**Conclusions:**

Almost two-thirds of middle-aged premenopausal participants showed low or moderate compliance with SENC recommendations. Education, smoking, parity, and physical activity were associated with the degree of adherence to these recommendations.

## Introduction

When studying different climacteric stages, it is often remarked the impact that perimenopause and menopause has on women's quality of life, but the premenopausal period is frequently forgotten on this matter. Premenopause is a natural period in a woman's adult life defined as the phase prior to perimenopause or the menopausal transition (1). There are studies showing that the clinical manifestations of the menopause can appear early, even before menstrual irregularities (2).

The fact that many middle-aged premenopausal women begin to show a clinical decline during the period close to menopausal transition, leads us to emphasize the need to assess the health status of these women before the onset of menopause. Although therapeutic intervention may be necessary, certain lifestyles, such as diet and physical activity, can help reduce many typical symptoms. The Mediterranean diet can be a useful tool in the management of menopause related obesity (3), and can improve cardiovascular risk factors, bone mineral density, mood, symptoms of depression, and prevent cognitive decline (4).

Diet quality is affected by a large number of factors, not only sex and age but also education, occupation, income level, and conventional indexes of socioeconomic status (5, 6). Studying the sociodemographic characteristics of women associated with dietary patterns is, therefore, very helpful to understand the inequalities of health and as it is currently of great interest in the nutritional epidemiology field (7). The study of the nutritional status of middle-aged women approaching menopause, as well as the associated factors or lifestyles, can help prevent or mitigate climacteric symptoms and improve the quality of life of these women.

Although several studies have been published that associate dietary intake with various menopausal symptoms (advising a high consumption of fruits, vegetables, whole grains, low-fat foods and products with high content in vitamin D) as a recent review suggests (8), these recommendations are very general and do not inform on the frequency and weight of daily servings. In 2019, the Spanish Society of Community Nutrition (SENC) updated its Food-Based Dietary Guidelines (9). These guidelines are based on the current dietary model of the Spanish population and include the Healthy Eating Pyramid, recommended serving sizes, practical recommendations for designing menus, purchasing, storing, and cooking food (10). Although there are previous studies that have evaluated the degree of compliance with the SENC dietary recommendations in the Catalan (11) and Balearic (12) population, in Spanish men and women from the EPIC (13), ANIBES (14), and SUN (15) studies, as well as in adolescents (16) and in women before and during pregnancy (17), none of them have focused on evaluating the factors associated with adherence to these recommendations in women approaching menopause.

The objective of this study is to evaluate the diet quality and the adherence to the SENC dietary recommendations in middle-aged Spanish premenopausal women, and to explore the sociodemographic and lifestyle factors that influence such adherence.

## Materials and Methods

### Study Population and Data Collection

DDM-Madrid is a cross-sectional study that recruited, between June 2013 and May 2015, a total of 1466 premenopausal women (39–50 years) that attended the Madrid City Council Medical Diagnostic Center (Madrid Salud), where they attended for their occupational gynecological examination. Women were invited to participate by phone before their medical appointment, and 88% agreed to collaborate. The study was conducted in accordance with the Declaration of Helsinki guidelines and was approved by the Ethics and Animal Welfare Committee of the Institute of Health Carlos III. All participants signed an informed consent form.

Trained interviewers administered a face-to-face structured computerized epidemiological questionnaire to participants, which included sociodemographic information, work history, tobacco smoking and alcohol consumption. Recreational physical activity during the previous year was measured using a translation of a validated questionnaire that considers duration, frequency, and intensity of 26 activities (18). Total metabolic equivalents (MET-h/week) were calculated according to the 2011 Compendium of Physical Activities (19). In addition, weight and height of the participants were measured using a certified bioimpedance scale (Tanita SC-330). After measuring each variable twice, with a third measure if the first two were discrepant, the mean values were used in the analyses.

Participants also answered a 117-item semi-quantitative food frequency questionnaire (FFQ) that has been validated in the Spanish adult population (20). It gathered information on eating habits for the previous 12 months, specifying standard portions or servings through 9 frequency categories (from “never or less than once a month” to “6 or more times a day”). The data was collected through an Access form and exported to Excel. The mean daily intake of some selected edible nutrients was calculated using the food composition tables of the U.S. Department of Agriculture (21), and other published tables for Spanish foods (22). The nutritional results as well as the data collected in all questionnaires were exported to STATA/MP 15.1 software.

Caloric and lipid profile was obtained according to the ranges recommended by Moreiras et al. (23), and the percentage of women at risk of insufficient intake were described following the European Food Safety Authority (EFSA) recommendations (24).

Subsequently, a score was created to assess the degree of compliance with the dietary recommendations for healthy eating proposed by the SENC (9, 10). Supplementary Table 1 shows the FFQ foods included in the score. When the participant complied with the recommendation of a food group, we assigned a score of 1, and when she did not comply, we assigned a score of 0. The final score was obtained as the sum of the individual scores. Therefore, our score ranged from 0 (when none of the recommendations were met) to 15 (when all were met).

After excluding 190 women who did not complete the FFQ, 13 participants for implausible dietary data, 5 participants who followed a vegetarian diet, and 7 without information on covariates, the final sample size included 1251 participants (Supplementary Figure 1).

### Statistical Methods

Descriptive analyses of women's characteristics, food groups, and energy and micronutrient intake were carried out using medians and interquartile ranges (IQR) for continuous variables, and absolute figures and percentages for categorical variables.

Participants were categorized into three groups, according to the tertiles of the SENC global adherence score: low adherence (<6), moderate adherence (6), and high adherence (7–12). Baseline women's characteristics by level of adherence to the SENC recommendations were described with total figures and percentages, and significant differences between the three levels of adherence (low, moderate and high) and these characteristics were tested with Pearson's chi-square tests.

The association between the three categories of adherence and lifestyle and sociodemographic variables was assessed using an ordinal multivariable logistic regression model. Proportional odds assumption was tested using the Brant test. This model was adjusted for educational level plus those variables that were associated with adherence to the SENC recommendations in the previous analysis (*p* <0.20). Analyses were performed using STATA/MP 15.1 software.

## Results

Diet quality according to the nutrient intake of the women is summarized in Tables 1, 2. Regarding caloric profile (distribution of macronutrients and alcohol as a percentage of total energy), most of the women exceeded the recommendations for caloric intake from proteins and fats, while caloric intake from carbohydrates was lower than recommended (i.e., were under 50%) for 92% of the participants. The consumption of saturated fat was higher than recommended, while the consumption of monounsaturated fats was lower in a large percentage of our participants (90.2% and 79.9%, respectively) (Table 1).

**Table 1:** Caloric profile and lipid profile^a^.

		**n**	**%**
**Caloric profile**			
Protein	10–15%^a^	39	3.1%
	>15%	1212	96.9%
Fat	<30%^a^	135	10.8%
	30–35%^a^	360	28.8%
	>35%	756	60.4%
Carbohydrates	<50%	1149	91.8%
	50–60%^a^	97	7.8%
	>60%^a^	5	0.4%
Alcohol	<10%^a^	1238	99.0%
	>10%	13	1.0%
**Lipid profile**			
Monounsaturated fat	<20%	999	79.9%
	≥20%^a^	252	20.1%
Polyunsaturated fat	<5%	234	18.7%
	≥5%^a^	1017	81.3%
Saturated fat	<8%^a^	122	9.8%
	>8%	1129	90.2%

a *Range Recommended According to Moreiras et al. (23)*.

**Table 2:** Micronutrient daily intake and percentage of women at risk of insufficient intake.

			**Risk of insufficient intake ^b^**	
	**RI^**a**^**	**Median (IQR)**	***n***	**%**
			**<** **80% RI**	
Calcium (mg)	950	1043.1 (816.0–1361.4)	245	19.6%
Iron (mg)	16	14.0 (11.0– 17.5)	489	39.1%
Iodine(μg)	150	353.7 (274.1–452.1)	6	0.5%
Zinc (mg)	10	12.8 (10.3– 15.7)	109	8.7%
Magnesium (mg)	300	350.7 (285.5–421.4)	150	12.0%
Potassium (mg)	3500	3348.4 (2709.6–4047.8)	359	28.7%
Vitamin B6 (mg)	1.6	2.1 (1.6– 2.8)	111	8.9%
Folate (μg)	330	288.7 (227.8–363.2)	499	39.9%
Cyanocobalamin (μg)	4	8.7 (6.5– 11.9)	19	1.5%
Vitamin C (mg)	95	135.5 (94.8–193.7)	197	15.7%
Vitamin A: Equivalents of retinol (μg)	650	982.7 (710.9–1360.3)	114	9.1%
Vitamin D (μg)	15	3.4 (2.6– 4.7)	1239	99.0%
Vitamin E (mg)	11	11.8 (8.7– 15.4)	318	25.4%

*IQR, interquartile range.*

a *RI, recommended intake according to EFSA 2019 (24).*

b *Risk of insufficient intake of micronutrient is considered when consumption is <80% of the RI*.

Table 2 shows the median micronutrient intake and the percentage of women at risk of insufficient intake. The most generalized deficient intake was observed for vitamin D (99.0%), followed by far by folate (39.9%), iron (39.1%), and potassium (28.7%).

Table 3 shows the score for the degree of adherence to the SENC recommendations by food groups, their distribution, and the median daily and weekly intake by food groups. Most women did not comply with the daily recommended amounts of olive oil (94.1%), fruits (88.1%), vegetables (79.2%), cereals, potatoes, pulses and others (73.3%) and dairy products (54.1%). On the other hand, the recommendation for water intake was highly met (71.6%). Regarding weekly recommendations, the worst adherences were found for sweets (97.2%), red meat, processed meat and other fats (95.4%), eggs (92.9%), nuts (75.6%) and white meat (65.8.0%), while a high adherence was observed for snacks (97.9%), alcohol (92.2%), legumes (81.4%) and fish (73.4%).

**Table 3:** Score and adherence to the SENC food–based dietary recommendations.

**Daily intake recommendations**	**Number of servings**	**Score**	**n**	**%**	**Daily intake (g or ml)**
					**Median (IQR)**
Water	<4 daily servings	0	355	28.4%	1141.8 (732.3–1641.8)
	**4–6 daily servings** ^**a**^	1	846	67.6%	
	**>6 daily servings** ^**a**^	1	50	4.0%	
Cereals,Potatoes, pulses and others	<4daily servings	0	874	69.9%	172.0 (112.9–229.7)
	**4–6 daily servings** ^**a**^	1	334	26.7%	
	>6 daily servings	0	43	3.4%	
Dairy products	<2 daily servings	0	500	40.0%	314.4 (217.9–555.4)
	**2–3 daily servings** ^**a**^	1	575	46.0%	
	>3 daily servings	0	176	14.1%	
Fruits	<3 daily servings	0	1102	88.1%	259.1 (157.7–397.7)
	**3–4 daily servings** ^**a**^	1	118	9.4%	
	**>4 daily servings** ^**a**^	1	31	2.5%	
Vegetables	<2 daily servings	0	991	79.2%	276.6 (196.5–376.9)
	**2–3 daily servings** ^**a**^	1	242	19.3%	
	**>3 daily servings** ^**a**^	1	18	1.4%	
Olive oil	<3 daily servings	0	1177	94.1%	11.0 (8.6– 27.5)
	**3–4 daily servings** ^**a**^	1	74	5.9%	
**Weekly intake recommendations**	**Number of servings**	**Score**	***n***	**%**	**Daily intake (g or ml)**
					**Median (IQR)**
Fish	<3 servings a week	0	333	26.6%	73.5 (51.8–106.1)
	**3–4 servings a week** ^**a**^	1	484	38.7%	
	**>4 servings a week**	1	434	34.7%	
White meat	<3 servings a week	0	517	41.3%	38.6 (18.9– 45.3)
	**3 servings a week** ^**a**^	1	428	34.2%	
	>3 servings a week	0	306	24.5%	
Eggs	<3 units per week	0	1162	92.9%	21.6 (7.2– 21.6)
	**3–5 units per week** ^**a**^	1	89	7.1%	
Legumes^b^	<2 servings a week	0	233	18.6%	22.1 (20.1– 60.2)
	**2–4 servings a week** ^**a**^	1	603	48.2%	
	**>4 servings a week** ^**a**^	1	415	33.2%	
Nuts	<3 servings a week	0	863	69.0%	4.3 (2.0– 12.9)
	**3–7 servings a week** ^**a**^	1	305	24.4%	
	>7 servings a week	0	83	6.6%	
Snacks	**<** **1 servings a week**^**a**^	1	1225	97.9%	15.8 (8.6– 28.2)
	≥ 1 servings a week	0	26	2.1%	
Alcoholic drinks	**<** **1.5 daily servings**^**a**^	1	1154	92.2%	33.5 (0.0–102.6)
	≥ 1.5 daily servings	0	97	7.8%	
Red/processed meat and other fats	**<** **1 servings a week**^**a**^	1	58	4.6%	81.7 (54.0–114.2)
	≥ 1 servings a week	0	1193	95.4%	
Sweets	**<** **1 servings a week**^**a**^	1	35	2.8%	59.0 (33.1–105.2)
	≥ 1 servings a week	0	1216	97.2%	

*IQR, interquartile range; SENC, Spanish society of community nutrition.*

a *Range recommended and servings sizes according to the SENC society (9).*

b *Including soy products and derivates (only the amount of soy in the product has been taken into account to quantify the servings)*.

Table 4 shows the distribution of sociodemographic and life-style characteristics by tertiles of adherence to the SENC recommendations. The overall score ranged from 2 to 12, with a median of 6.0 (IQR: 5.0-7.0). The percentage of women with low, moderate, and high adherence was 40.5%, 27.8% and 31.7%, respectively. In general, low adherence was more frequent among nulliparous, smokers, and those who consumed fewer calories.

**Table 4:** Characteristics of the women, overall and by tertiles of the SENC adherence score.

			**Adherence**			
			**Low**	**Moderate**	**High**	
			**Tertile 1 (2–5 points)**	**Tertile 2 (6 points)**	**Tertile 3 (7–12 points)**	
			**507 (40.5%)**	**348 (27.8%)**	**396 (31.7%)**	
	***N* = 1251**	**%**	***n* (%)**	***n* (%)**	***n* (%)**	***p*–value^**a**^**
Age (years)^b^						
39–43	519	41.5%	213 (41.0)	138 (26,6)	168 (32.4)	0.652
44–46	425	34.0%	176 (41.4)	114 (26.8)	135 (31.8)	
47–50	307	24.5%	118 (38.4)	96 (31.3)	93 (30.3)	
Educational level						
Primary school or less	52	4.2%	23 (44.2)	18 (34.6)	11 (21.2)	0.450
Secondary school	431	34.5%	174 (40.4)	124 (28.8)	133 (30.9)	
University graduate	768	61.4%	310 (40.4)	206 (26.8)	252 (32.8)	
Body mass index (Kg/m^2^)						
<25	857	68.5%	356 (41.5)	221 (25.8)	280 (32.7)	0.100
25–29	287	22.9%	104 (36.2)	97 (33.8)	86 (30.0)	
≥30	107	8.6%	47 (43.9)	30 (28.0)	30 (28.0)	
Number of children						
None	303	24.2%	136 (44.9)	88 (29.0)	79 (26.1)	0.055
1	278	22.2%	119 (42.8)	66 (23.7)	93 (33.5)	
≥2	670	53.6%	252 (37.6)	194 (29.0)	224 (33.4)	
Tobacco smoking						
Never	489	39.1%	200 (40.9)	145 (29.7)	144 (29.4)	0.045
Former smoker	441	35.3%	160 (36.3)	122 (27.7)	159 (36.1)	
Current smoker	321	25.7%	147 (45.8)	81 (25.2)	93 (29.0)	
Living with a partner						
No	292	23.3%	126 (43.2)	84 (28.8)	82 (28.1)	0.316
Yes	959	76.7%	381 (39.7)	264 (27.5)	314 (32.7)	
Following a diet						
No	856	69.2%	342 (40.0)	233 (27.2)	281 (32.8)	0.420
Yes	381	30.8%	158 (41.5)	112 (29.4)	111 (29.1)	
Total energy intake (kcal/day)						
<1708.5	417	33.3%	273 (65.5)	88 (21.1)	56 (13.4)	<0.001
1708.5–2188.2	417	33.3%	156 (37.4)	137 (32.9)	124 (29.7)	
>2188.2	417	33.3%	78 (18.7)	123 (29.5)	216 (51.8)	
Eating out						
Never or <3 times a month	784	63.2%	307 (39.2)	219 (27.9)	258 (32.9)	0.634
1–4 times a week	401	32.3%	171 (42.6)	109 (27.2)	121 (30.2)	
>4 times a week	55	4.4%	25 (45.5)	16 (29.1)	14 (25.5)	
Occupational qualification^c^						
Low	139	11.1%	53 (38.1)	48 (34.5)	38 (27.3)	
Medium	691	55.3%	281 (40.7)	182 (26.3)	228 (33.0)	0.364
High	420	33.6%	173 (41.2)	117 (27.9)	130 (31.0)	
Physical activity (METS –h/week)						
<3	551	44.0%	218 (39.6)	168 (30.5)	165 (29.9)	0.135
3–9	220	17.6%	91 (41.4)	50 (22.7)	79 (35.9)	
10–19	216	17.3%	95 (44.0)	63 (29.2)	58 (26.9)	
≥20	264	21.1%	103 (39.0)	67 (25.4)	94 (35.6)	

*SENC, Spanish society of community nutrition.*

a *P–value from Pearson's chi–square tests.*

b *In tertiles.*

c *According to the 9 major occupational sectors of the 2011 National classification of Occupations (CNO−11). High qualification: 1–3, Medium qualification: 4–6, Low qualification: 7–9 (25)*.

The adjusted association between adherence to the SENC score and several sociodemographic and lifestyle factors is shown in Table 5. Former smokers (OR: 1.38; 95%CI: 1.08–1.78), as well as those with higher educational level (OR_secondary_:1.68; 95%CI: 0.97–2.93, OR_university_:1.82; 95%CI: 1.05–3.14), with two or more children (OR: 1.31; 95%CI: 1.00-1.72), with higher caloric intake (OR_<2188.2kcal/day_: 8.22; 95%CI: 6.19–10.92) and with greater physical activity (OR_≥21METS−h/week_: 1.29; 95%CI: 0.95–1.76) showed greater adherence to the SENC score, while those with higher body mass index (BMI) (OR_≥30_: 0.77; 95%CI: 0.52–1.15) presented a lower degree of adherence.

**Table 5:** Association between adherence to SENC recommendations and potential influential factors.

**Variable**	**OR^**a**^**	**(95% CI)**	**Homogeneity^**b**^**
Educational level			0.095
Primary school or less	1.00		
Secondary school	1.68	(0.97–2.93)	
University graduate	1.82	(1.05–3.14)	
Body mass index (Kg/m^2^)			0.318
<25	1.00		
25–29	1.08	(0.83–1.40)	
≧30	0.77	(0.52–1.15)	
Number of children			0.145
None	1.00		
1	1.20	(0.87–1.67)	
≧2	1.31	(1.00–1.72)	
Tobacco smoking			0.005
Never	1.00		
Former smoker	1.38	(1.08–1.78)	
Current smoker	0.90	(0.68–1.19)	
Total energy intake (kcal/day)^c^			<0.001
<1708.5	1.00		
1708.5–2188.2	3.16	(2.41–4.15)	
>2188.2	8.22	(6.19–10.92)	
Physical activity (METS–h/week)^d^			0.191
Sedentary	1.00		
<9	1.21	(0.90–1.62)	
9–21	0.94	(0.70–1.27)	
> 21	1.29	(0.95–1.76)	

*SENC, Spanish society of community nutrition.*

a *Odds ratio adjusted for all variables included in the table.*

b *Test for homogeneity of adherence across categories of each variable.*

c *In tertiles.*

d *In tertiles of physically active participants*.

## Discussion

This study tries to evaluate the quality of the diet in middle-aged Spanish premenopausal women, the degree of compliance with the SENC food-based dietary guidelines, and to explore the sociodemographic and lifestyle factors that are associated. Overall, 41% of participants showed low compliance with these recommendations, while 32% showed high compliance. More than 85% of the women did not consume the daily or weekly recommended amounts either due to a lack of olive oil, fruit and eggs, or due to an excess of sweets, red or processed meat. Practically all of participants had an insufficient intake of vitamin D. Most women exceeded the recommendations for caloric intake from proteins and fat (mainly saturated fat). Former smokers, those with a higher educational level, with two or more children or with higher caloric intake showed greater adherence to the SENC dietary recommendations.

The results on daily servings consumed are similar to those detected by Úbeda et al. in Spanish menopausal women (26). These authors -taking the 2004 SENC recommendations as reference (27) found an insufficient intake of fruits and nuts, as well as a high consumption of meat and its derivatives and sweets. However, and unlike our results, the alcohol consumption of their participants was higher than that recommended by the SENC (10). On the other hand, when comparing the mean consumption in grams with that of the nationally representative sample of the Spanish women population of the ANIBES study, the intake of dairy products, vegetables and fruits is lower in our participants compared to theirs; the consumption of water and eggs is higher, and that of olive oil and alcohol is very similar (28).

Considering the caloric profile, we have observed that the energy intake from fats and proteins is above current SENC recommendations, while the intake from carbohydrates (fruits, vegetables, cereals, potatoes, and legumes) is below. These results are in line with the evolution observed in the Spanish diet over the last fifty years, with a decrease in the contribution of carbohydrates to the diet and an increase of fats (29). Since fat accumulation and redistribution often occur during the climacteric stage, the contribution of high-fat foods at this stage of life should be carefully examined (30). A distinction must also be made between foods rich in polyunsaturated fats from those rich in saturated fats, since the first group is beneficial for the prevention of some degenerative diseases, and it has been described that they can improve the symptoms derived from menopause (31). Although the consumption of polyunsaturated fat was adequate in 81% of our participants, the consumption of saturated fat was excessive in 90% of them. The main sources of saturated fatty acids in the diet of the Spanish population are milk and dairy products, meat and its derivatives, and oils and fats (32). Several studies have associated the consumption of saturated fats with an increase in the intensity of symptoms related to menopause or with depressive symptoms in middle-aged women (8). Regarding monounsaturated fats (MUFA), their proportional contribution to the overall caloric intake was lower than that recommended by Moreiras et al. (23) in most of our women, contrary to what was observed in the previously mentioned study by Varela-Moreiras et al. (29). This last result may be due to the lower consumption of olive oil (main source of MUFAs in the Mediterranean diet) observed in our participants.

Micronutrient intake was, with some exceptions, mostly in accordance with EFSA recommendations. The main exception was vitamin D intake, which was lower than recommended in 99% of participants. This is consistent with the fact that vitamin D deficiency is very frequent in Spain, involving approximately 40% of the adult population (33). Vitamin D is necessary for intestinal calcium absorption and bone and muscle homeostasis, and can help reduce menopausal-related symptoms and improve sexual function and bone health in perimenopausal women (34). However, it should be noted that the most important source of vitamin D is sun exposure, and that only a modest quantity can be obtained from foods such as fatty fish, dairy products or eggs (35). We also found intakes of iron, folate and potassium below the recommended levels. These micronutrients must be carefully monitored over the years to detect possible negative trends early.

Compliance with the SENC recommendations was higher in women with higher educational level, in former smokers, in those with higher caloric intake, in those having two or more children and, to a lesser extent, in those with higher physical activity. The greater adherence in people with higher educational level is a fairly consistent finding in Europe, mainly in relation to fruits and vegetables consumption (36). It has been described that higher educational level can be related to increased competencies, skills and knowledge, which are important for engaging with health education messages and avoiding harmful behaviors (37). The higher cost of healthy diets has also been associated with lower adherence to these diets in population groups with lower educational level (38, 39).

The better adherence detected in women with children is consistent with that reported by other authors (40). Regarding grocery shopping, it has been observed that women with children tend to focus more on nutrition and the preferences of children, while women without children pay more attention to the taste and the preferences of other household members. Women without children have also been found to eat more often in fast food chains (41).

On the other hand, the greater adherence observed in former smokers may be explained by the fact that the acquisition of a healthy lifestyle (such as quitting smoking) can promote the acquisition of other healthy behaviors, such as improved diet or physical activity. In this sense, in a randomized controlled trial on different strategies to quit smoking, Berg et al. observed that reducing and quitting smoking was associated with higher intake of fruits and vegetables and more walking for exercise at 26 week follow-up (42).

Regarding total energy intake, its increase is usually associated with an increase in nutrient intake (43). However, a study that evaluated the diet quality in a representative Spanish population, showed that low energy density diets are associated with a healthier lifestyle and tend to be closer to the SENC dietary recommendations than high-energy density diets (44). Another study conducted in the elderly population, showed that meeting nutrient intake recommendations is difficult with low energy consumption, even in the case of diets based on the Mediterranean dietary pattern (45). A good diet should therefore achieve a greater richness in nutrients with a low energy intake (always within the recommendations).

Finally, the greater adherence detected in participants with greater physical activity has also been reported in previous studies, in which a positive association was observed with adherence to a “Spanish-Mediterranean” diet (46), Mediterranean diet (40) and other health conscious diets (44). In general, people with higher level of physical activity tend to be healthier than less active people, and tend to consume a more varied diet, while sedentary people consume more fast food products and fewer fruits and vegetables, which responds to less healthy eating profiles (47, 48). Regarding the SENC dietary recommendations in particular, lower adherence has been observed in Spanish adolescents with low physical activity (16), as well as greater adherence to the SENC food pyramid among the most physically active participants of the SUN cohort (15).

Our study has several strengths. Firstly, to our knowledge, this is the first study examining sociodemographic and lifestyle determinants associated with adherence to the SENC dietary recommendations in premenopausal women. We also count with a large sample size, a high participation rate and standard and validated tools to collect the data.

However, our study also has several limitations. First, it is a cross-sectional study, so it is not possible to establish causal relationships between the sociodemographic/lifestyle variables and compliance with the SENC recommendations. Second, these variables were self-reported, which implies a potential source of information bias. Third, these women were recruited from a single center in Madrid and, although they came from all over the province, this limits the ability to generalize the study results. With regard to dietary assessment, data were collected using FFQs, which did not prevent potential measurement errors and may suffer from inaccuracies. However, the use of FFQs still remains a widely used tool for dietary assessment in epidemiological studies (49). Finally, it is necessary to mention the limitations inherent to the calculation of the score of adherence to the SENC recommendations, such as the categorization of food intake into dichotomies of “compliance” and “non-compliance,” or the possible misclassification of some food groups (50, 51).

In conclusion, our results show that the degree of adherence to the SENC recommendations in middle-aged Spanish premenopausal women was, in general, low to moderate. Some recommendations should be promoted, mainly those related to a higher consumption of olive oil and eggs and a lower consumption of sweets, red meat, and processed meat. The consumption of vitamin D and fats, especially saturated fats, should be in accordance with the recommendations. In addition, adherence was better among more educated women, women with children, former smokers, and more physically active participants. It would be advisable to promote campaigns to disseminate the SENC recommendations, both to the general public and to this group of women in particular, as well as launching actions that promote healthier eating habits, with diets focused on preventing or mitigating climacteric symptoms and, ultimately, improve the quality of life of this group of women who are approaching menopause.

## Data Availability Statement

Data described in the article are not publicly available due to restrictions imposed by the Institute of Health Carlos III Ethics Committee, but are available from the principal investigator on reasonable requests. To access the datasets requests should be directed to ER-M: e.ruiz@externos.isciii.es.

## Ethics Statement

The study was reviewed and approved by the Ethics and Animal Welfare Committee of the Institute of Health Carlos III. The patients/participants provided their written informed consent to participate in this study.

## Author Contributions

MP, VL, and ER-M designed research. PL, AS, and BP-G conducted research. MP and MM-C provided essential materials. MA-C, IA-L, AC, VL, and ER-M analyzed data. MA-C, VL, ER-M, and NFL-B wrote the paper. MP, VL, and ER-M had primary responsibility for final content. All authors have read and approved the final manuscript.

## Funding

This study was funded by the Spanish Ministry of Health (EC11–273) and by the Instituto de Salud Carlos III (PI15CIII/0029). The article presents independent research.

## Author Disclaimer

The views expressed are those of the authors and not necessarily those of the Institute of Health Carlos III.

## Conflict of Interest

The authors declare that the research was conducted in the absence of any commercial or financial relationships that could be construed as a potential conflict of interest.

## Publisher's Note

All claims expressed in this article are solely those of the authors and do not necessarily represent those of their affiliated organizations, or those of the publisher, the editors and the reviewers. Any product that may be evaluated in this article, or claim that may be made by its manufacturer, is not guaranteed or endorsed by the publisher.

## Abbreviations

EFSA: European Food Safety Authority
FFQ: Food frequency questionnaire
MET: Total metabolic equivalents
MUFAs: Monounsaturated fatty acids
OR: Odds ratio
REDLINC: Collaborative Group for Research of the Climacteric in Latin America (Red Latinoamericana de Investigación en Climaterio).
