# Supplementary material for: Sociodemographic and Lifestyle Determinants of Adherence to Current Dietary Recommendations and Diet Quality in Middle-Aged Spanish Premenopausal Women
Source: Front Nutr. 2022 Jun 14;9:904330. doi: 10.3389/fnut.2022.904330 (PMC9237508; doi:10.3389/fnut.2022.904330)
Supplement: Supplementary file 1 [file Data_Sheet_1.docx]

Supplementary Material

**Table of Contents:**

**Supplemental Table 1.** FFQ foods included in the score for the adherence to the SENC recommendations.

**Supplemental Figure 1.** Flowchart of selecting participants

**Supplemental Table 1**. FFQ foods included in the score for the adherence to the SENC recommendations.

| Food groups recommended by SENC | FFQ Food included |
| --- | --- |
| Water | Water |
| Cereals, Potatoes, pulses and others | Bread (white, whole), rice, pasta, breakfast cereals, potatoes, wheat germ |
| Dairy products | Milk (whole, skimmed, enriched/fortified), yogurt (whole, skimmed), cheese (cured, semi-cured, cream) cottage cheese |
| Fruits | Fruits and natural fruits juices |
| Vegetables | Vegetables |
| Olive oil | Olive oil |
| Fish | Fish, shellfish, canned and smoked fish and shellfish |
| White meat | Chicken, turkey, duck, rabbit, quail |
| Eggs | Eggs |
| Legumes ^2^ | Lentils, chickpeas, black or white beans, soy milk*, soy yogurt* |
| Nuts | Nuts |
| Snacks | Olives, french fries,  potato chips |
| Alcoholic drinks | Wine, beer and sider |
| Red meat, processed meats and others fats | Beef, pork, lamb, cold meat, ham, sausages, bacon, pâtés, other vegetable oils, butter and margarine |
| Sweets | Croissant, Donut, muffin, cake or similar, cookies, pastry, chocolate, powder, cocoa, sugar, jams, honey |

* Only the amount of soy in the product has been taken into account to quantify the servings.

**Supplemental Figure 1**. Flowchart of selecting participants in the DDM-Madrid study

**2144 potentially eligible**

Women (39–50 years) who attended the Madrid City Council Medical Diagnostic Centre (Madrid Salud), between June 2013 and May 2015

**1466 eligible**

Premenopausal women interviewed

**678 excluded**

57 untraceable

426 not eligible

195 refused to participate

99,282 women without pregnancy, or with twins only from 1991-2001

**1251 included**

**215 excluded**

190 not completed the FFQ

13 implausible dietary data

5 vegetarian diet

7 without information on covariates
